# A Phase I, Randomized, Double-Blinded, Placebo- and Moxifloxacin-Controlled, Four-Period Crossover Study To Evaluate the Effect of Gepotidacin on Cardiac Conduction as Assessed by 12-Lead Electrocardiogram in Healthy Volunteers

**DOI:** 10.1128/AAC.02385-16

**Published:** 2017-04-24

**Authors:** Mohammad Hossain, Meijian Zhou, Courtney Tiffany, Etienne Dumont, Borje Darpo

**Affiliations:** a RD Projects Clinical Platforms & Sciences, GSK, King of Prussia, Pennsylvania, USA; b RD Projects Clinical Platforms & Sciences, GSK, Collegeville, Pennsylvania, USA; c RD Infectious Disease, GSK, Collegeville, Pennsylvania, USA; d Karolinska Institute, Department of Clinical Sciences, Danderyd's Hospital, Division of Cardiovascular Medicine, Stockholm, Sweden; e iCardiac Technologies, Rochester, New York, USA

**Keywords:** QT prolongation, QTc, cardiac safety, healthy subjects, thorough QT study

## Abstract

Gepotidacin is a novel, first-in-class triazaacenaphthylene antibiotic in development for treatment of conventional and biothreat infections. This was a single-dose, crossover thorough QT study in healthy subjects who were administered intravenous (i.v.) gepotidacin as a therapeutic (1,000-mg) dose and supratherapeutic (1,800-mg) dose, placebo, and 400 mg oral moxifloxacin in 4 separate treatment periods. Gepotidacin caused a mild effect on heart rate, with a largest placebo-corrected change-from-baseline heart rate of 7 and 10 beats per minute at the end of the 1,000-mg and 1,800-mg infusion, respectively. Gepotidacin caused an increase of change-from-baseline QTcF (ΔQTcF), with a peak effect at the end of infusion. The largest mean placebo-corrected ΔQTcF (ΔΔQTcF) was 12.1 ms (90% confidence interval [CI], 9.5 to 14.8) and 22.2 ms (90% CI, 19.6 to 24.9) after 1,000 mg and 1,800 mg, respectively. ΔΔQTcF rapidly fell after the end of the infusion, with a mean ΔΔQTcF of 6.1 ms 60 min after the 1,800-mg dose. Exposure-response analysis demonstrated a statistically significant positive relationship between gepotidacin plasma levels and ΔΔQTcF, with a slope of 1.45 ms per μg/ml (90% CI, 1.30 to 1.61). Using this model, the effect on ΔΔQTcF can be predicted to be 11 and 20 ms at the observed mean peak plasma concentration after the infusion of gepotidacin at 1,000 mg (7 μg/ml) and 1,800 mg (13 μg/ml), respectively. In conclusion, gepotidacin caused QT prolongation in this thorough QT study, and a mean effect can be predicted to less than 15 ms at the highest expected plasma concentration, 9 μg/ml. (This study has been registered at ClinicalTrials.gov under identifier NCT02257398.)

## INTRODUCTION

Gepotidacin, a first-in-class novel triazaacenaphthylene bacterial topoisomerase inhibitor, inhibits bacterial DNA replication and has *in vitro* activity against key pathogens, including drug-resistant strains associated with a range of conventional and biothreat infections. Gepotidacin selectively inhibits bacterial DNA replication by interacting in a unique way with the GyrA subunit of bacterial DNA gyrase and the ParC subunit of bacterial topoisomerase IV. This interaction appears to be highly specific to bacterial topoisomerases, as evidenced by weak inhibition of human topoisomerase IIα, supporting the selective activity of gepotidacin against the bacterial target. As a consequence of its novel mode of action, gepotidacin is active *in vitro* against target pathogens resistant to established antibacterials, including fluoroquinolones. Gepotidacin is available as oral and intravenous (i.v.) formulations and is currently being evaluated in phase II studies for acute bacterial skin and skin structure infections (ABSSSI) and gonorrhea (GC).

A potential for QT prolongation was identified in nonclinical studies with gepotidacin. The drug inhibited the human ether-à-go-go-related gene (hERG) ion tail current with a 50% inhibitory concentration (IC_50_) of 588 μg/ml, which is 96-fold greater than the highest anticipated clinical free-fraction plasma concentration (*C*_max_) of 6.1 μg/ml after an i.v. dose of 1,000 mg three times daily (TID), based on 33% plasma protein binding in human (GSK, unpublished data). In an *ex vivo* rabbit left ventricular wedge preparation, gepotidacin caused a concentration-dependent increase in the QRS interval, moderate QT prolongation, and an increase of transmural dispersion of repolarization at ≥135 μg/ml, 22-fold greater than the highest anticipated free-fraction *C*_max_. A torsadogenic potential was also noted in this assay at 67 μg/ml, approximately 11-fold greater than the highest anticipated clinical *C*_max_ (GSK, unpublished data). In an *in vivo* cardiovascular study with i.v. gepotidacin in cynomolgus monkeys, moderate and reversible increases in heart rate, arterial blood pressure, and an index of cardiac contractility were observed at the highest tested dose (250 mg/kg of body weight), with a 7-fold exposure margin versus clinical concentrations. Mild QTc prolongation (4% to 9%) was also noted at 1.2- to 7.0-fold the anticipated highest clinical *C*_max_ (6.1 μg/ml), as well as a widening of the QRS interval (GSK, unpublished data). Furthermore, in a meta-analysis of phase I data, QTc prolongation was seen in healthy subjects following i.v. doses which resulted in high plasma concentrations of gepotidacin. It was therefore considered important to conduct this thorough QT (TQT) study in parallel with phase II studies while maintaining cardiovascular monitoring in all subjects receiving gepotidacin.

This study was designed to meet the requirement for a TQT study, as defined in the ICH E14 document (1) with subsequent clarifications through the question and answers document (2). Peak plasma levels after an i.v. infusion of gepotidacin are higher than those after oral dosing; therefore, this route of administration was chosen for this study, with the intention of achieving supratherapeutic plasma levels. However, adverse events related to cholinergic effects, which have been attributed to acetylcholinesterase inhibition by the drug, have been observed after gepotidacin infusion (3). This effect seems to be related to *C*_max_ but not to the exposure (area under the concentration-time curve [AUC]), and it seems to be mitigated by maintaining plasma concentrations of gepotidacin below 14 μg/ml. A 1,800-mg i.v. dose given as a 2-h infusion achieves this objective, had acceptable tolerability in previous phase I studies, and therefore was used as the supratherapeutic dose in this TQT study.

## RESULTS

Fifty-five subjects with a mean age of 31 years (range, 18 to 55) and mean body mass index (BMI) of 26 kg/m^2^ (range, 19.9 to 30.7) were enrolled into the study. Twenty-seven subjects (49%) were females, and the majority were white (*n* = 39; 71%) or black or African American (*n* = 11; 20%).

**FIG 1:**
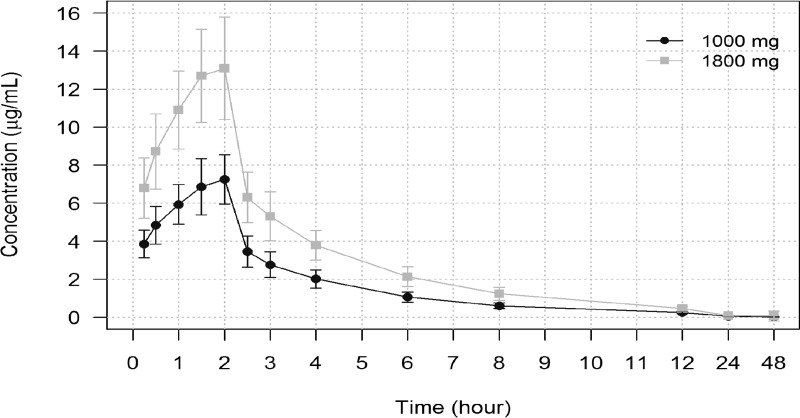
Plasma concentration-time course after an intravenous infusion of 1,000 mg and 1,800 mg gepotidacin. Means ± SD are shown.

Gepotidacin plasma concentration-time profiles are provided by treatment in Fig. 1. Mean gepotidacin plasma concentrations increased steadily during the 2-h i.v. infusion and then declined in a multiexponential fashion after the end of the infusion. The gepotidacin concentrations were generally detectable in plasma up to 48 h after the start of infusion.

At the predosing baseline, there were data from 50 to 53 subjects in each treatment period. Electrocardiogram (ECG) parameters were well balanced across predose baseline time points, with mean heart rates (HR) between 60.6 beats per minute (bpm) and 62.1 bpm, mean QTcF between 401.7 ms and 402.8 ms, mean PR between 146.2 ms and 148.6 ms, and mean QRS between 103.5 ms and 104.0 ms.

**FIG 2:**
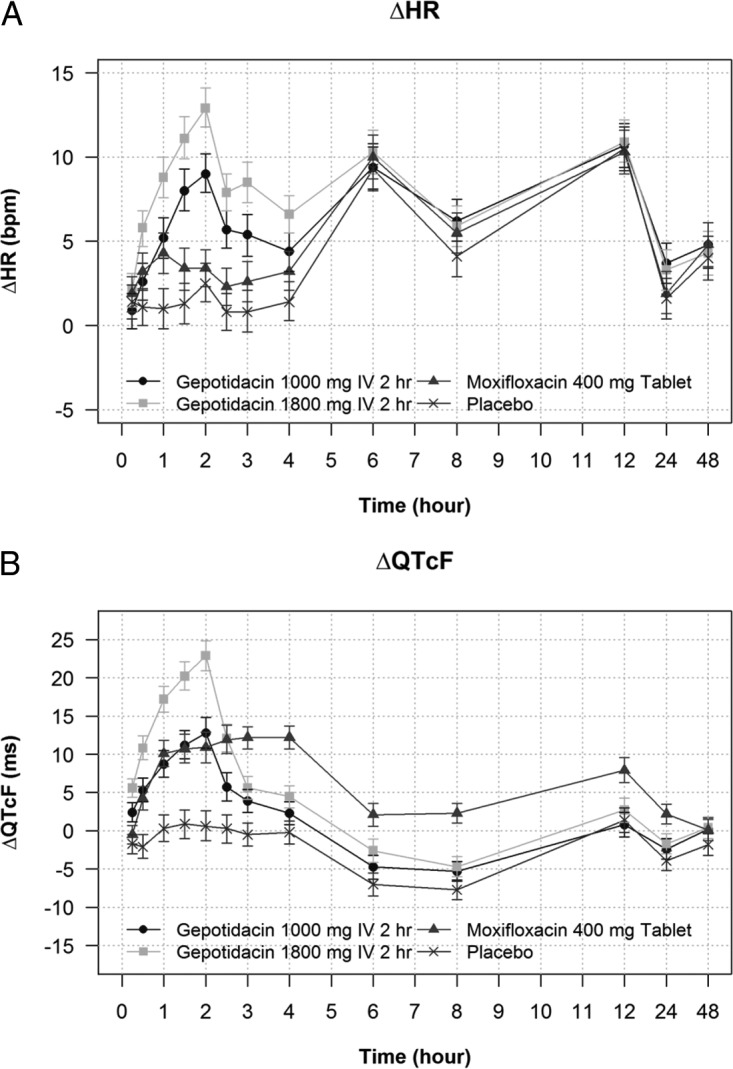
(A) Change-from-baseline heart rate (ΔHR) across treatments and time points. Least-squares means and 90% CI from the statistical modeling are shown. (B) Change-from-baseline QTcF (ΔQTcF) across treatments and time points. Least-squares means and 90% CI from the statistical modeling are shown.

The 2-h infusion of gepotidacin caused an increase of change-from-baseline HR (ΔHR), which peaked at the end of the infusion (2 h) at 9.0 bpm (90% CI, 7.9 to 10.2) after 1,000 mg and at 12.9 bpm (90% CI, 11.8 to 14.1) after 1,800 mg (Fig. 2A). At time points later than 4 h after the start of the infusion, the diurnal pattern of ΔHR was the same in all treatment periods. The placebo-corrected ΔHR (ΔΔHR) reached a largest mean value of 6.5 bpm (90% CI, 5.1 to 7.8) and 10.4 bpm (90% CI, 9.1 to 11.7), observed at the end of the 1,000-mg and 1,800-mg infusion, respectively.

Since the largest ΔΔHR exceeded 8 bpm, QTci and QTcI were derived. The mean slope of QTcI, which was calculated from all QT/RR during 24 h at baseline, was 0.3219 (standard deviations [SD], 0.067), i.e., close to the correction factor for Fridericia (0.33) (4). The mean slope for QTci, derived from supinely resting data on the day before dosing, was 0.3872 (SD, 0.051), i.e., substantially steeper and therefore closer to the QTcB (0.5) (5). When the ability of each correction method (QTcF, QTci, and QTcI) to remove the heart rate dependence of the QTc interval was tested using the mean of squared individual slopes (SSS), the lowest on-treatment SSS was observed with QTcF and QTcI, with clearly larger values with QTci (Table 1). QTcF was selected as the primary endpoint, since it consistently produced somewhat lower values than QTcI (0.0017 versus 0.0018 on placebo and 0.0049 versus 0.0066 on 1,800 mg gepotidacin).

**TABLE 1:** Average sum of squared slopes for different heart rate correction methods for QTc across treatments^*a*^

Treatment	Slope estimate (mean of squared individual slopes)		
	QTcF	QTcI	QTci
Gepotidacin 1,000 mg i.v. 2 h	0.0023	0.0025	0.0038
Gepotidacin 1,800 mg i.v. 2 h	0.0049	0.0066	0.0092
Moxifloxacin 400-mg tablet	0.0024	0.0024	0.0022
Placebo	0.0017	0.0018	0.0016

a As proposed in the publication by Tornoe et al. (6).

Gepotidacin caused an increase of change-from-baseline QTcF (ΔQTcF), which evolved during and peaked immediately after the end of infusion (2 h), with a ΔQTcF of 12.8 ms (90% CI, 10.8 to 14.8) after 1,000 mg and 22.9 ms (90% CI, 20.9 to 24.8) after 1,800 mg (Fig. 2B). The largest placebo-corrected ΔQTcF (ΔΔQTcF) was also observed at the end of the infusion (Table 2), with 12.1 ms (90% CI, 9.5 to 14.8) after 1,000 mg and 22.2 ms (90% CI, 19.6 to 24.9) after 1,800 mg gepotidacin (Table 2). The mean peak ΔΔQT effect was somewhat larger in females than in males: 13.4 versus 11.1 ms after the 1,000-mg gepotidacin dose and 25.6 versus 19.5 ms after the 1,800-mg dose. After the end of the infusion, ΔΔQTcF fell rapidly (Table 2) with a mean ΔΔQTcF of 11.9 ms 30 min later (2.5 h) and 6.1 ms at 3 h; all mean values from 4 h onwards were below 5 ms in the 1,800-mg gepotidacin treatment period (Table 2). Results from the analysis of QTcI (data not shown) were very similar to those for QTcF.

**TABLE 2:** Placebo-corrected change-from-baseline QTcF^*a*^

Time point (h)	ΔΔQTcF (ms) for^*b*^:		
	Gepotidacin 1,000 mg i.v.	Gepotidacin 1,800 mg i.v.	Moxifloxacin 400 mg (ms)
0.25	4.1 (2.6; 5.7)	7.3 (5.7; 8.9)	1.2 (−0.4; 2.8)
0.5	7.4 (5.4; 9.5)	12.9 (10.9; 14.9)	6.3 (4.3; 8.3)
1	8.4 (6.1; 10.7)	16.9 (14.6; 19.2)	9.8 (7.4; 12.1)
1.5	10.4 (7.9; 12.9)	19.4 (16.9; 21.9)	9.8 (7.3; 12.3)
2	12.1 (9.5; 14.8)	22.2 (19.6; 24.9)	10.3 (7.6; 12.9)
2.5	5.5 (3.0; 7.9)	11.9 (9.5; 14.3)	11.7 (9.3; 14.1)
3	4.4 (2.4; 6.3)	6.1 (4.2; 8.0)	12.7 (10.7; 14.6)
4	2.5 (0.5; 4.4)	4.6 (2.7; 6.6)	12.4 (10.5; 14.3)
6	2.3 (0.3; 4.3)	4.4 (2.5; 6.4)	9.2 (7.2; 11.1)
8	2.4 (0.7; 4.1)	3.0 (1.4; 4.7)	10.0 (8.3; 11.6)
12	−0.6 (−2.8; 1.5)	1.3 (−0.8; 3.4)	6.5 (4.4; 8.7)
24	1.5 (−0.1; 3.2)	2.2 (0.5; 3.8)	6.0 (4.4; 7.7)
48	2.0 (0.1; 3.9)	2.2 (0.3; 4.0)	1.9 (0.1; 3.7)

a Results from the linear mixed-effects model. Primary endpoints were used.

b Results are least-squares means with 90% CI in parentheses.

Assay sensitivity was confirmed by the observed QT prolongation after oral dosing of 400 mg moxifloxacin. The largest mean ΔΔQTcF was observed at 3 h (12.7 ms), with the lower bound of the 90% CI being above 5 ms at all prespecified time points (7.6 ms, 10.7 ms, and 10.5 ms at 2, 3, and 4 h, respectively) (Table 2).

**FIG 3:**
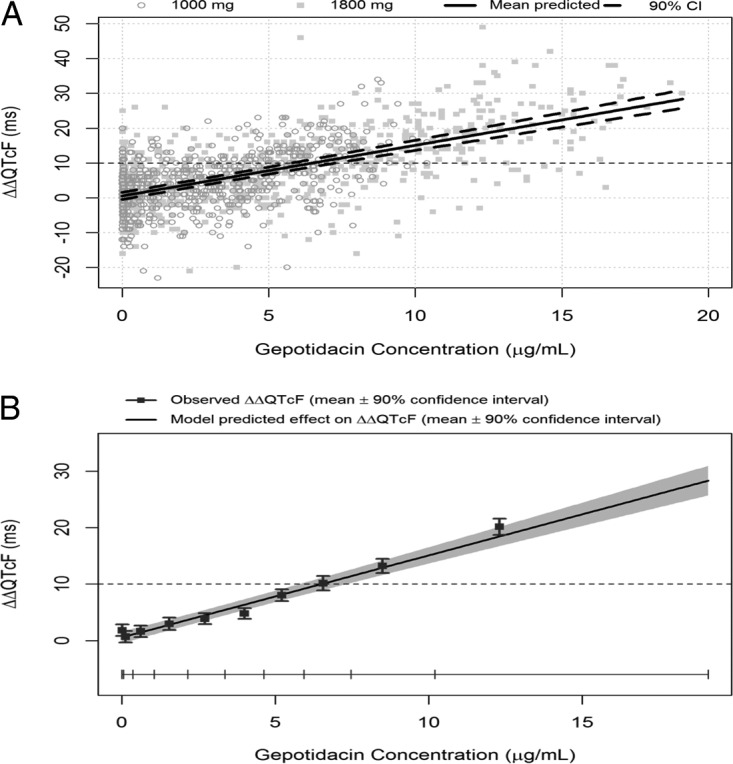
Relationship between gepotidacin plasma concentrations and placebo-corrected change-from-baseline QTcF (ΔΔQTcF). (A) Scatter plot with all observed ΔΔQTcF/plasma concentration pairs and exposure-response model predicted effect (red line) with 90% CI. (B) Exposure-response model-predicted ΔΔQTcF (means and 90% CI) and observed ΔΔQTcF (means with 90% CI) within each gepotidacin plasma concentration decile.

In the exposure-response analysis, a linear model with an intercept provided a good fit to the data (Fig. 3A and B). A concentration-dependent effect of gepotidacin on the QTcF interval (ΔΔQTcF) was identified with a slope of the relationship of 1.45 ms per μg/ml (90% CI, 1.30 to 1.61) and an intercept of 0.55 ms (90% CI, −0.50 to 1.60). Using the exposure-response model, the mean effect on ΔΔQTcF can be predicted to be 11.2 ms (90% CI, 10.0 to 12.4) and 19.9 ms (90% CI, 18.0 to 21.7) at the observed geometric *C*_max_ after the 1,000-mg (7.3 μg/ml; 90% CI, 7.02 to 7.66) and 1,800-mg (13.3 μg/ml; 90% CI, 12.72 to 13.85) dose, respectively. When gender was analyzed as part of the exposure-response (ER) model, the slope of the plasma concentration/ΔΔQTc relationship was somewhat smaller in females than in males (1.38 ms per μg/ml versus 1.54 ms per μg/ml), but the observed *C*_max_ was higher, 14.8 versus 12.0 μg/ml, after the 1,800-mg gepotidacin dose. The predicted QT effect at the observed *C*_max_ was therefore comparable: 20.6 ms in females versus 19.2 ms in males.

Infusion of gepotidacin had a small shortening effect on the PR interval, which coincided with the observed HR effect; the largest mean effect after the 1,800-mg dose of gepotidacin was observed at 2 h, with a ΔΔPR of −7.1 ms (90% CI, −9.0 to −5.1). Gepotidacin did not have a clinically relevant effect on the QRS interval; the largest ΔΔQRS was only 1.2 ms (90% CI, 0.8 to 1.5), observed 1.5 and 2 h after the start of the 1,800-mg infusion.

### Safety.

Adverse events were reported by 23 subjects (46%) after 1,000 mg gepotidacin and by 34 subjects (64%) after 1,800 mg gepotidacin. They were generally mild to moderate in severity and included nausea, abdominal pain, abdominal discomfort, vomiting, salivary hypersecretion, feeling hot, and oropharyngeal discomfort. After administration of gepotidacin, the moderate adverse events were vomiting (8 subjects [15%]), nausea (4 subjects [8%]), throat tightness (1 subject [2%]), and dizziness (1 subject [2%]), all after the 1,800-mg dose. Five subjects (9%) discontinued the study due to adverse events, including one case of throat tightness, 3 positive tests for Clostridium difficile, and one case of C. difficile infection.

A sensitivity analysis excluding subjects who experienced vomiting after the 1,800-mg gepotidacin dose did not materially change the results: the largest QT effect (mean ΔΔQTcF) excluding these subjects was 21.4 ms, observed at 2 h, compared to 22.2 ms in the full population. Cholinergic side effects such as salivary hypersecretion were few and mild, and it seems unlikely that these affected the QT analysis.

## DISCUSSION

This TQT study demonstrated that gepotidacin caused QTc prolongation with a largest mean effect on ΔΔQTcF of ∼13 ms after a dose of 1,000 mg and ∼23 ms after 1,800 mg, given as an i.v. dose over 2 h. The peak effect was observed immediately after the end of the infusion and thereafter fell rapidly. The QTc effect was linearly related to plasma concentrations of the drug with a statistically significant slope of the relationship of 1.45 ms per μg/ml (90% CI, 1.30 to 1.61). A moderate heart rate effect of the drug was noted with a mean peak effect of approximately 10 to 13 bpm at the end of 1,000-mg and 1,800-mg infusions. The observation of QTc prolongation at high gepotidacin plasma levels is consistent with the nonclinical findings, and it confirms and further qualifies the observation made for the pooled phase I data.

Using these results as a basis, it is possible to gain insight and quantify the level of QT prolongation that can be expected in patients given different doses and formulations of gepotidacin. Based on the established exposure-response relationship from this TQT study, it can be predicted that the mean QT effect (ΔΔQTcF) in patients administered an oral dose of 1,500 mg will be around 7.5 ms (90% CI, 6.8 to 8.3 ms). Based on a short half-life of the drug, repeat dosing will lead to only small (9% to 18%) increases of *C*_max_; however, plasma concentrations may be higher in patients with impaired clearance of the drug due to, for example, drug interactions. Gepotidacin is a CYP3A4 substrate; therefore, the potential for drug interaction was evaluated in a study with itraconazole, a known strong inhibitor of CYP3A4 and a P-glycoprotein inhibitor. A therapeutically relevant dose of gepotidacin, 1,500 mg, was administered to healthy subjects as a single dose and then again after 3 days of dosing with 200 mg itraconazole. During maximum 3A4 inhibition on itraconazole, the geometric means of *C*_max_ and AUC_0–∞_ for gepotidacin were approximately 40% to 50% greater than those for gepotidacin alone, indicating a weak drug interaction (GSK, unpublished data). Concomitant administration of gepotidacin and a strong CYP3A4 inhibitor is thought to represent the worst-case scenario in terms of plasma levels in patients on oral therapy. A 40% increase of C_max_ in patients on 1,500 mg orally would lead to levels around 6.7 μg/ml, and the mean QT effect can then be estimated to ∼10 ms (10.3 ms; 90% CI, 9.3 to 11.3). Intravenous dosing regimens result in higher gepotidacin plasma concentrations. In patients with ABSSSI, the highest i.v. dose that has been explored in phase 2 studies to date is 1,000 mg infused over 2 h TID. The observed geometric mean *C*_max_ was 8.9 μg/ml after this i.v. dose and 8.6 μg/ml after the corresponding oral dose of 2,000 mg TID in the same study. The QT effect at these plasma concentrations can be predicted to ∼13 ms (13.4 and 13.0 ms), with an upper bound of the 90% CI below 15 ms (14.8 and 14.4 ms).

Many macrolides and fluoroquinolone antibiotics have been shown to cause QTc prolongation. The QT effect of moxifloxacin has been well characterized through its use in several hundred TQT studies, and a therapeutic oral dose of 400 mg causes QTc prolongation of between 10 and 16 ms (7–10), an effect level that seems comparable with the expected QT effect at the highest therapeutic gepotidacin i.v. dose (1,000 mg; ∼9 μg/ml). Macrolides have been associated with QT prolongation and torsades de pointes (TdP) proarrhythmias in susceptible patients, and erythromycin, clarithromycin, and azithromycin are all listed on the Credible Medicines website as drugs with known risk of torsades de points (11). QT prolongation at the level that can be expected in patients on high therapeutic doses of gepotidacin, i.e., below 15 ms, is thought to carry a very low risk of proarrhythmias. However, it should be noted that higher plasma levels, and therefore a somewhat higher QT effect, may be seen in patients with impaired clearance of the drug due to intrinsic or extrinsic factors. Gepotidacin will be administered to patients in whom first-line therapy has failed or who are intolerant to available antibiotics. Given the medical need of these patients and with appropriate cautionary measures in place, such as correction of hypokalemia before initiation of therapy and exclusion of patients on other drugs known to cause QT prolongation, treating these patients with gepotidacin seems well justified. Furthermore, gepotidacin is an antibiotic that may serve as an important medical countermeasure against drug-resistant biothreat pathogens. As far as we know, gepotidacin remains the only new antibiotic to demonstrate efficacy in the FDA-accepted animal model of inhalational plague (Yersinia pestis). It is also the most advanced and only antibiotic with a novel mechanism able to address multiple other clinically important bioterror agents and therefore may become an important future societal medical countermeasure to protect against the release of resistant biothreat agents.

In conclusion, infusion of gepotidacin at doses of 1,000 mg and 1,800 mg over 2 h caused QTcF prolongation of 12 ms and 22 ms, respectively, at the end of the infusion, with a rapidly declining effect thereafter. Based on the exposure-response (QTc) relationship observed in this study, QT prolongation of approximately 13 ms (with an upper bound of the 90% CI of 15 ms) can be predicted at the highest achieved mean plasma levels in patients, i.e., around 9 μg/ml.

## MATERIALS AND METHODS

This TQT study was randomized and conducted in healthy subjects using a 4-period, actively and placebo-controlled, double-blind, crossover design. Subjects received all study drugs in separate treatment periods: placebo, gepotidacin at 1,000 mg and 1,800 mg i.v., and oral moxifloxacin at 400 mg. The primary objective was to evaluate the effect of single i.v. doses of 1,000 mg and 1,800 mg gepotidacin on the heart rate-corrected QT interval as determined by the change-from-baseline QTc (ΔQTc) compared with the placebo. A double-dummy approach was used to maintain blinding, i.e., on each dosing day, moxifloxacin or moxifloxacin placebo and i.v. gepotidacin or matched i.v. placebo was administered. In the first treatment period, subjects were admitted to the clinical research unit 2 days before dosing (day −2) and remained at the clinical research unit until completion of the last safety assessment on day 3. In the remaining periods, subjects were admitted on the day before dosing. Fifty-five healthy subjects, using standard clinical pharmacology criteria, were to be randomized to ensure 46 evaluable subjects completed the study.

Study treatments (A through D) were given in randomized sequence in separate periods: A, 1,000 mg gepotidacin i.v. over 120 min and oral moxifloxacin placebo; B, 1,800 mg gepotidacin i.v. over 120 min and oral moxifloxacin placebo; C (placebo), gepotidacin placebo i.v. over 120 min and oral moxifloxacin placebo; D (positive control), oral moxifloxacin at 400 mg and gepotidacin placebo i.v. over 120 min.

The therapeutic dose, 1,000 mg i.v. as a 2-h infusion, is the highest dose anticipated to be used in phase III clinical studies. The selection of the highest gepotidacin dose, 1,800 mg i.v. as a 2-h infusion, was based on cholinergic adverse effects previously observed with higher concentrations when 1,800 mg was given as a 1-h infusion. Therefore, it was well understood that achieved peak plasma levels with this dose would not generate truly supratherapeutic plasma levels, typically more than 3- to 6-fold above clinically relevant concentrations (2). Oral moxifloxacin (400 mg) was chosen as the positive control to demonstrate assay sensitivity. Moxifloxacin has been well characterized in numerous TQT studies (7), and the criteria for demonstration of assay sensitivity are based on an expected mean QTc prolongation of ∼8 to 15 ms, typically seen in these studies (see question 3.1 in reference 2). A minimum 7-day washout separated each treatment period. A follow-up visit was conducted 7 to 10 days after day 3 of the final dosing period.

### Cardiodynamic ECG assessment.

Continuous 12-lead ECGs were recorded for 48 h starting 1 h before dosing in all treatment periods using a Global Instrumentation (Manlius, New York, USA) M12R ECG continuous 12-lead digital recorder. A 24-h ECG recording also was performed on the day before dosing in period 1 to allow for derivation of individualized QTc methods if a substantial heart rate effect was to be observed. Subjects were resting in the supine position during 15 min before and 5 min after each prespecified time point for ECG extraction. ECGs were extracted in up to 10 replicates at the following time points: at 3 time points predose (45, 30, and 15 min prior to starting the infusion), at 0.25, 0.5, 1.0, 1.5, 2.0 (end of infusion), 2.5, 3.0, 4.0, 6.0, 8.0, 12.0, 24, and 48 h after dosing, and at corresponding nominal time points on the day before dosing in period 1. Standard procedures were followed at the central ECG laboratory (iCardiac Technologies, Rochester, NY), which included that ECG analysts were masked to the subject, visit, and treatment allocation and that baseline and on-treatment ECGs for a particular subject were overread on the same lead by the same reader. Measurements of ECG intervals were performed using the high-precision QT technique. Up to 10 replicate ECGs were extracted at each time point, and QT and RR interval measurements were made initially by the underlying ECG algorithm, COMPAS, as previously described (12). All beats that were deemed high confidence based on criteria including heart rate stability and ECG pattern were measured by the computerized algorithm, while all other beats were overread and either accepted without adjustment or rejected. With the HPQT technique, up to 120 beats are measured per time point. The primary analysis lead was lead II. If it was not analyzable, then the primary analysis lead was changed to another lead for the entire subject data set.

### Statistical analyses.

Based on the observation of QTc prolongation at high intravenous concentration in phase I studies, an estimation approach was used, aimed to quantify the magnitude of the effect on QTc from single i.v. doses of gepotidacin.

All statistical analyses were performed using the statistical software SAS for Windows, version 9.3 (SAS Institute, Inc., Cary, NC). The mean from the 3 predose values (45, 30, and 15 min prior to infusion) was used as the baseline for each postdosing time point in the same period. For the placebo adjustment in the exposure-response (ER) analysis, the individual change from baseline for QTc (ΔQTc) on placebo calculated at a specific time point was subtracted from ΔQTc for the same subject on gepotidacin at the same time point to generate the placebo-corrected ΔQTc (ΔΔQTc).

The primary endpoint was ΔQTcF. If a substantial heart rate effect was observed on treatment with gepotidacin, the primary endpoints were to be selected based on 3 different correction methods' abilities to remove the heart rate dependence of QTc based on prospectively defined criteria. Secondary endpoints included change-from-baseline heart rate (ΔHR), PR (ΔPR), QRS (ΔQRS), categorical outliers for ECG intervals, and treatment-emergent T-wave morphology changes, as well as the relationship between gepotidacin plasma levels and the effect on the QTc interval and the safety and tolerability of i.v. gepotidacin following a single 1,000-mg and 1,800-mg dose.

In case the largest mean effect of gepotidacin on the placebo-corrected ΔHR exceeded 8 bpm, two variants of an individualized heart rate correction of QTc were to be derived from drug-free data on the day before dosing of period 1, in addition to QTcF. QTci was derived from QT/RR pairs extracted from the time points at which subjects were supinely resting on the day before dosing. QTcI was derived from all QT/RR pairs from the full 24-h recording. The QT/RR pairs from each subject were used for that subject's individual correction coefficient, derived from a linear regression model: log(QT) = log(α) + β × log(RR). The coefficient of log(RR) for each subject, βi, was then used to calculate the individually corrected QT for that subject with the following equation: QTc = QT/RR^βi^. The relationship between QTc (QTcF, QTci, and optimized QTcI) and RR interval then was investigated using on-treatment data (gepotidacin at 1,000 mg and 1,800 mg and placebo) by linear regression modeling: QTc = *a* + *b* × RR. The RR coefficient for each subject, βi, was then used to calculate the average sum of squared slopes (SSS) for each of the different QT-RR correction methods, using the method proposed by members of the FDA's Interdisciplinary Review Team for QT studies (6). The correction method that resulted in the slope closest to zero for on-treatment data was deemed the most appropriate HR correction method and therefore was used for the primary endpoint.

The primary analysis for gepotidacin was based on a linear mixed-effects model with ΔQTcF as the dependent variable and with time (categorical), treatment (gepotidacin at 1,000 mg and 1,800 mg, moxifloxacin, and placebo), and time-by-treatment interaction as factors and baseline QTcF as a covariate. Since this was a crossover design, period and sequence terms were also included in the model. Subject was included as a random effect for the intercept. Gender effect was added in the model for exploration; if period, sequence, and gender effects were not statistically significant at the alpha level of 0.05, they were excluded from the final model. An unstructured covariance matrix was specified for the repeated measures at postdose time points for the subject within-treatment period. For this analysis, the least-squares means and 90% confidence intervals were calculated for the contrast, termed gepotidacin − placebo. The same linear mixed-effects model was also applied to those QTc methods that have not been selected as the primary endpoint and to HR, PR, and QRS to compute the mean change between gepotidacin and placebo and the corresponding 90% CI.

The analysis to demonstrate assay sensitivity was based on ΔQTc on moxifloxacin. The model described for the primary analysis was used. For the time points of 2, 3, and 4 h, the contrast in treatment, ΔΔQTc = moxifloxacin − placebo, was tested against the one-sided null hypothesis of a ΔΔQTc of ≤5 ms on the 5% level. Multiplicity was controlled by using a Hochberg procedure (13). If, after this procedure, ΔΔQTc was significantly larger than 5 ms for at least one time point, assay sensitivity was considered shown. In addition, 2-sided 90% CIs were obtained for the contrast at all time points for descriptive purposes and was used in the figures.

The relationship between ΔΔQTcF and plasma concentrations of gepotidacin was investigated by a linear mixed-effects modeling approach, for which 3 models were considered: (i) a model with an intercept, (ii) a model with mean intercept fixed to 0 (with variability), and (iii) a model with no intercept. Time-matched concentration was included in the model as a covariate, and subject as a random effect for both intercept and slope whenever applicable. For diagnostic purposes, a plot of standardized residuals versus fitted values was used to examine departure from model assumptions. The normal Q-Q plots of the random effects and the within-subject errors were used to investigate the normality of the random effects and the within-subject errors, respectively. A final assessment of the adequacy of the linear mixed-effects model was provided by a goodness-of-fit plot (6). Via visual inspection of the goodness-of-fit plot, the assumption of linearity between ΔΔQTcF and plasma concentrations of gepotidacin and how well the predicted ΔΔQTcF matched the observed data in the regions of interest were checked. The linear exposure-response model that fit the data best (i.e., had the smallest Akaike information criterion and had predicted CIs similar to the observed CIs) was used to evaluate the exposure-response relationship.
